# Numerical biomechanics modelling of indirect mitral annuloplasty treatments for functional mitral regurgitation

**DOI:** 10.1098/rsos.211464

**Published:** 2022-01-12

**Authors:** Lee Galili, Adi White Zeira, Gil Marom

**Affiliations:** School of Mechanical Engineering, Tel Aviv University, Tel Aviv 6997801, Israel

**Keywords:** mitral valve, indirect mitral annuloplasty (IMA), numerical models, biomechanics, cardiovascular devices

## Abstract

Mitral valve regurgitation (MR) is a common valvular heart disease where an improper closure leads to leakage from the left ventricle into the left atrium. There is a need for less-invasive treatments such as percutaneous repairs for a large inoperable patient population. The aim of this study is to compare several indirect mitral annuloplasty (IMA) percutaneous repair techniques by finite-element analyses. Two types of generic IMA devices were considered, based on coronary sinus vein shortening (IMA-CS) to reduce the annulus perimeter and based on shortening of the anterior–posterior diameter (IMA-AP). The disease, its treatments, and the heart function post-repair were modelled by modifying the living heart human model (Dassault Systèmes). A functional MR pathology that represents ischaemic MR was generated and the IMA treatments were simulated in it, followed by heart function simulations with the devices and leakage quantification from blood flow simulations. All treatments were able to reduce leakage, the IMA-AP device achieved better sealing, and there was a correlation between the IMA-CS device length and the reduction in leakage. The results of this study can help in bringing IMA-AP to market, expanding the use of IMA devices, and optimizing future designs of such devices.

## 1. Introduction

Mitral valve regurgitation (MR) is a common valvular disease with an estimated five million incidences worldwide [1]. In this disease, blood leaks from the left ventricle (LV) into the atrium because of improper valve closure during systole. In functional MR (FMR), the malfunction is not of the valve itself and is usually caused by LV dysfunction [2], where the motion of the papillary muscles is restricted, leading to less tension in the chordae and limited annular contraction during systole. A common cause of FMR is ischaemia of the left ventricle [3].

Although the standard treatment for severe FMR is open-heart surgery, it is contraindicated for a large population of inoperable patients [4]. Therefore, there is a need for alternative treatments, such as percutaneous repairs. The only United States Food and Drug Administration (FDA) approved percutaneous repair is the MitraClip (Abbott Laboratories, Chicago, IL, USA). This device mimics surgical edge-to-edge repair [4]. Other CE mark approved treatments are the Edwards Pascal system, which also mimics the edge-to-edge repair, Mitralign and Cardioband, which are based on direct mitral annuloplasty, and the Cardiac Dimensions Carillon System [5]. The latter is the only one that is based on indirect mitral annuloplasty (IMA), a reshaping of the annulus by anatomic adjustments not in the annulus itself, and it does not mimic a surgical technique.

The IMA device, the Carillon System, is based on a shortening of the coronary sinus (CS), a vein that is parallel and close to the mitral annulus [6]. This device has been implanted in approximately 1000 patients [5,7] and is indicated for treatment of FMR. It is built from two NiTi anchors with a bridge between them that are implanted in the CS. Thus, shortening the distance between the anchors reduces the annulus perimeter. The latest generation of the device demonstrated an improvement in MR reduction, left ventricular volumes and distinctly improved walk test results [8–10]. Another approach to IMA is to bring the anterior and posterior (AP) leaflets closer to each other by shortening the AP diameter of the annulus. While there are still no clinically approved devices that employ this approach, two such devices have been proposed, the Arto system (MVRx Inc., Belmont, CA) and the trans-apical segmented reduction annuloplasty (TASRA; MitraSpan Inc., Belmont, MA, USA) [11]. The Arto system is a percutaneous treatment that is still under development and is aimed at the treatment of FMR [12,13]. By connecting two anchors with a suture, one in the CS and the other in the atrial septum, the AP diameter is reduced [13,14]. A recent clinical study demonstrated that the device successfully decreases the symptoms of the disease after 1 year while also being safe [12]. Still, it is not clear how these two IMA approaches differ in their capabilities to reduce MR leakage.

Engineering methods can help in studying the leakage reduction mechanisms of such devices. Numerical modelling has many benefits when it comes to such complicated biomechanics, and it enables tests that cannot be done in *in vitro* or *in vivo* experiments [15]. Moreover, it is possible to isolate the disease conditions and examine the same treatment in several repairs of the same disease. Numerical models have been employed in previous studies to simulate healthy and diseased mitral valves and their surgeries [16–19]. Moreover, surgical repair and percutaneous treatments of MR have been previously modelled by finite-element analyses (FEA), including models of annuloplasty [20–24] and edge-to-edge [25–28] treatments to evaluate the effects of these procedures on tissue stress, tension in the chordae tendineae, and haemodynamics. Baillargeon *et al*. [29] presented a model of an annuloplasty ring to treat ischaemic MR. This model accounted for the fact that the valve malfunction was not a result of the valve itself but of the diseased myocardium. To achieve it, they used the living heart human model (LHHM; Simulia, Dassault Systèmes, Providence, RI, USA), an electro-mechanically coupled heart simulator of the entire heart with the electrophysiology and myocardial fibre architecture of a median adult male [30] that was modified to introduce the pathology. The same group later modelled the implantation of another annuloplasty ring that was designed to treat FMR [31]. There are several previous models of percutaneous MR treatment, most commonly the MitraClip [25,27]. Kamakoti *et al*. [32] modelled the haemodynamics after MitraClip repair by using the smoothed particle hydrodynamics (SPH) approach. They found that the leakage decreased when the clip was implanted closer to the missing chordae. Finally, while in a recent study by our group [33], we demonstrated the capability of the model to capture indirect annular reduction, the focus was on a device that pulls the chordae tendineae during its deployment process, rather than a device for MR treatment.

While several types of MR treatments have been modelled by FEA, no previous study has modelled IMA devices like the Carillon or Arto. Additionally, annuloplasty models focused on either rigid or highly compliant ring devices, and it was reasonable to neglect the effect of the heart beating on the device's configuration during the heart cycle. The present study aims to assess and compare the effects of two types of IMA treatments on post-procedural outcomes. Specifically, the aim is to study the effect of generic devices, which resemble the Carillon and Arto approaches, on the leakage reduction in FMR. In order to achieve these objectives, numerical simulations were used.

## 2. Methods

In this study, implantations of two generic IMA devices were modelled by three-dimensional FEA. The first device aims to shorten the annulus through the CS (IMA-CS), while the second device aims to shorten the AP diameter (IMA-AP), thus representing the functions of the Carillon and Arto devices, respectively. These implantations were simulated in a beating heart with FMR pathology, and several shortening distances were compared with the untreated case to estimate the effectiveness of the treatments.

**Figure 1.**
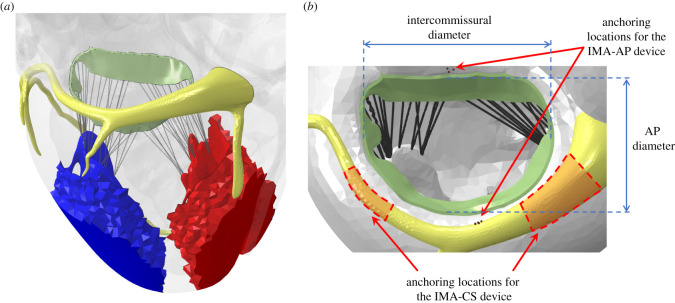
Schematic descriptions of the geometry of the model: (*a*) The left ventricle with the mitral valve marked in green, the coronary sinus vein in yellow, the posterior papillary muscles in red, and the anterior papillary muscles in blue. (*b*) A view of the mitral valve with a schematic representation of the anchoring locations for both types of indirect mitral annuloplasty (IMA) devices, through the coronary sinus (CS) vein and the anterior posterior (AP) diameter.

The heart model was based on the LHHM, and it was solved in Abaqus (Simulia). The subvalvular apparatus consists of four papillary muscles divided into AP groups, with two muscles in each one of them (figure 1*a*). Since the LHHM represents a healthy heart, ischaemic FMR pathology was reproduced here by following the methods suggested by Rausch *et al*. [31]. In this pathology, myocardial infarction disables the contraction in regions of the left ventricle, and it is common in the papillary muscles. Therefore, the material properties in the infarction region of the posterior papillary muscles were changed from being active to being passive throughout the cycle (figure 1*a*). The material properties of the infarction region were identical to the active tissue, except for an increased activation threshold that was set at 100 mV, above the maximum action potential in the sinoatrial (SA) node (20 mV). The infarction region included 44% of the elements in the posterior papillary muscles to ensure the mitral valve could not close during systole. To choose the infarcted elements, first all the posterior papillary muscle elements were deactivated, and then the number of elements was reduced until we found the minimal number of elements that still prevented the valve closure with a similar orifice area. In this model, as in the default LHHM, the AP and intercommissural diameters of the valve in the undeformed geometry (representing the diastole) are 34.4 and 35.9 mm, respectively (figure 1*b*), and the annular perimeter is 118.5 mm. The applied pressure load on the leaflets was prescribed by calculating the pressure difference between the blood pressures in the LV and the left atrium (LA). Unlike the original LHHM v. 2.1, a short phase shift of 50 ms was defined between the haemodynamic pressures in the LV and the atrium to the pressure load on the leaflets of the valve in all the models of the current study. This phase shift allowed better closure of the valve and it represents the physiologic shift between the atrium-LV pressure difference and the pressure exerted on the valve leaflets [34].

The first generic device, IMA-CS, was modelled as two anchors (see representative locations in figure 1*b*) with a bridge that connects them. For simplification, the surfaces of tube-like anchors were modelled and meshed by membrane elements and the wire that forms the bridge was meshed by beam elements. The anchors were almost rigid with linear elastic properties (Poisson's ratio of 0.35 and elasticity modulus of 100 GPa) and a thickness of 0.5 mm. The bridge was made of super-elastic NiTi with 14 material constants [35]. The device implantation in the CS vein was modelled when the anatomy was in a diastolic position, the relaxed heart anatomy before modelling the heart cycle. First, a tube was inserted into the CS by a predefined motion along the centreline of the vein, then the distal anchor was expanded, the bridge was shortened and moved towards the valve to reduce the annular perimeter, and finally, the proximal anchor was expanded, as shown in the supplementary animation (electronic supplementary material, 1). While expanding the anchors, the contact pushed the vein outwards and forced the vein to assume the shape of the anchors. All the steps of this predefined motion of the device were enforced as displacement boundary conditions that were calculated by an in-house code which was implemented in Matlab (Mathworks Inc., Natick, MA, USA) to represent a 14% shortening of the bridge. Two additional shorter lengths of the device's bridge, representing 18% and 22% shortening of the bridge, were attained by employing a thermal contraction (negative expansion) boundary condition. After reaching the final geometry of the repaired valves, the geometries of the heart and the device were used in models of three full cardiac cycles with the implanted devices. To enforce perfect anchorage of the device, the anchors were directly constrained to move with (tied to) the ventricles and the left atrium in the beating heart models. In each one of these models, the systolic position of the valve was examined to estimate if the procedure had successfully repaired the regurgitation.

The second generic device that was simulated, IMA-AP, has a similar concept to the Arto system. To model it, two groups of nodes in the CS and the atrial septum, marked in figure 1*b*, were displaced. These groups were selected so the nodes would connect several elements instead of imposing a local displacement. First, the distance and the direction between the two groups were measured and three different desired distances were imposed to represent a shortening of 30%, 50% and 70% of the initial distance. Similar to the previous device, this shortening simulation was done in a diastolic position. Then a surgical suture, which was modelled by truss elements with a cross-section area of 0.074 mm^2^ and with material properties of ePTFE [36], was constructed to connect the node groups in the CS and the LV locations of the modified geometries. The suture was connected to the heart with a distributed force coupling to smoothly disperse the tension load. As in the previous device, the beating heart was modelled with the repaired valve.

The success of the repair procedures was evaluated during peak systole, defined in this study as 75% of the simulated systolic step. The pathological case was compared with the three models of the IMA-CS and the three cases of the IMA-AP. The regurgitation was directly estimated from the systolic results by analysing the contact area and estimating the regurgitant orifice area (ROA). While the contact area is based on the locations of nodes that were exposed to contact forces, the ROA was estimated by an in-house Matlab code that finds the closest nodes to the opposite leaflet by searching for them along longitudinal lines on the leaflets and filtering out nodes that are in contact. These results will also be presented on a spread view of the valve to demonstrate the locations of possible leaks, as was previously suggested for clinical scans [37]. To achieve it, the results were analysed by an in-house Matlab code that first used disc conformal parametrization by the code of Choi & Lui [38], and then plotted the spread results according to the circumferential and radial coordinates as schematically shown in electronic supplementary material, 2.

**Figure 2.**
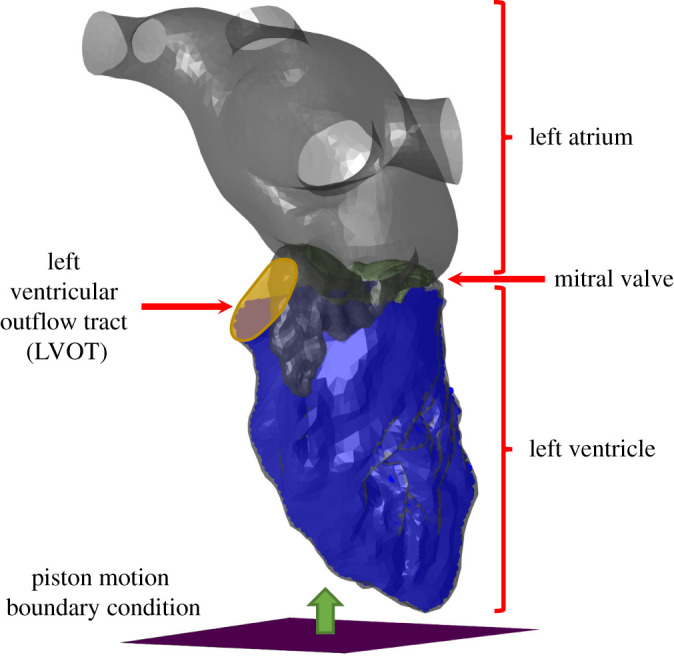
Schematic description of the smoothed particle hydrodynamics (SPH) models.

Finally, to quantify the blood leakage during systole, additional models of the regurgitant blood flow were carried out by employing the simplified SPH method, as commonly used for this type of leakage calculation in the mitral valve [18,28,32,36,39–42]. For simplification, only the flow in the left side of the heart was considered. In all the models, the same Cartesian mesh was used, but because of the difference between the geometries, the number of particles in the LV was different. Between 28 714 and 29 895 particles were seeded in the ventricle. These numbers of particles were chosen to ensure that there is a significant number of particles that leaked through the valve in the pathologic model (more than 1000), so a reduction of this number after the treatments will be meaningful. A piston motion was used to model the ventricular volume reduction, as shown in figure 2, while the LV, LA and the valve were fixed. The leakage through the mitral valve was quantified by counting the number of particles that reached the LA relative to the total number of particles that went out of the LV, including those that reached the aorta. Therefore, since all the particles have the same volume, this ratio represents the ratio between the volume of blood that leaked and the total volume of blood that went out of the LV because of the ventricular volume change. For that purpose, the location of each particle was identified as being either in the LV, LA or the aorta by an in-house code that compared their coordinates to the triangulated geometry of each volume.

## 3. Results

In this section, the results of the implantation models of the various device configurations are presented first. Then, the results of the beating heart models are presented to compare the function of these treatments and their capability to seal the valve during peak systole. Finally, the predicted regurgitations are compared based on the results of the leakage models.

### 3.1. Device implantation models

**Figure 3.**
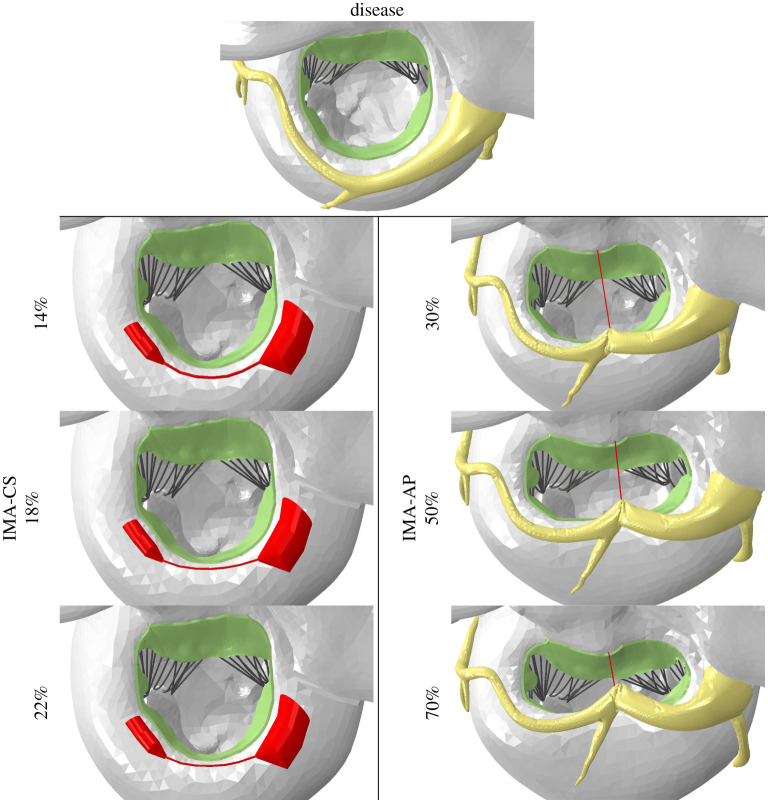
The various mitral valve configurations in their diastolic positions pre- and post-implantation of IMA-CS and IMA-AP devices.

First, the post-implantation configurations of the IMA-CS and IMA-AP devices were compared with each other and between the different sizes of each generic device. The implantation was modelled in the idealized diastolic position, before the simulation of the heart cycle. Figure 3 compares the diseased valve before repair relative to the post-implantation states of the various repairs. The use of IMA-AP led to a change in the valve's shape and brought the initial position of the AP leaflets closer to each other in the region of the device. For example, in the case of 70% shortening, the AP diameter was reduced from 34.4 to 14.3 mm in the disease and post-repair configurations, respectively. On the other hand, in the IMA-CS there are no visually distinguished changes in the shape or the size of the valve. This fact can also be demonstrated by the fact that a relatively large shortening of the bridge between the anchors, from 30 mm in the disease to 23.3 mm in the case of 22%, led to only a minor change in the intercommissural diameter, from 35.9 to 34.1 mm, respectively.

### 3.2. Post-implantation function: systolic configuration from a beating heart model

**Figure 4.**
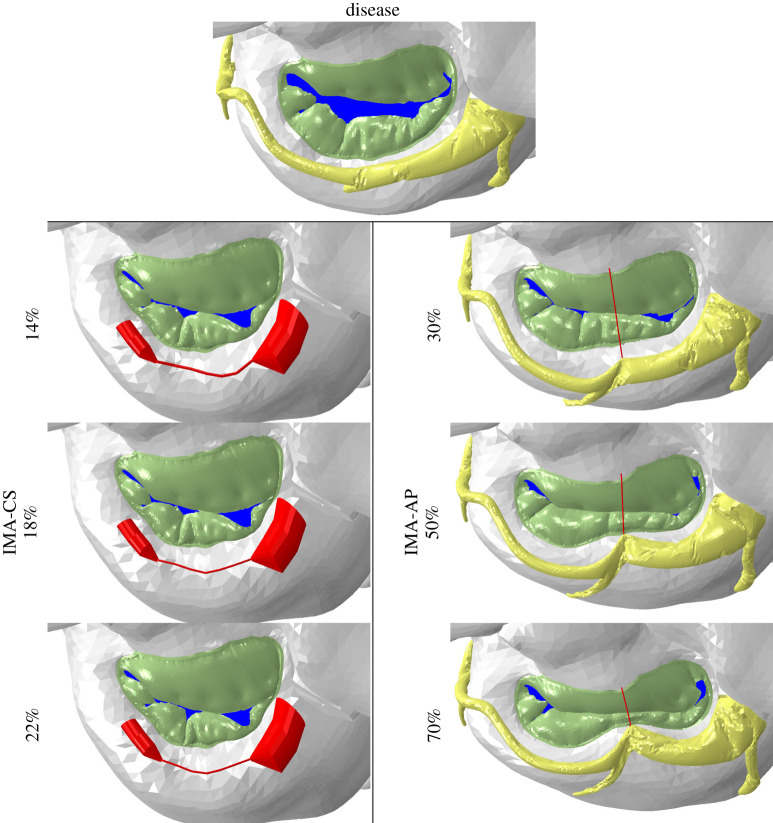
The various mitral valve configurations in their systolic positions pre- and post-implantation of IMA-CS and IMA-AP devices.

The results of the implantation models were then used to model the effect of the devices on the valve functionality during the heart cycle. The valve closures at peak systolic position are compared in figure 4. This figure also shows the location of the calculated ROA, which is marked in blue. It can be seen that the IMA-AP devices closed the valve better than the IMA-CS devices in all sizes. The area that remained unsealed in the IMA-AP devices is concentrated mainly in the commissures' region. On the other hand, while the IMA-CS devices reduce the ROA relative to the disease, there is a prolapse in the posterior leaflet that prevents them from getting an even better closure. These findings can also be seen by looking at the quantitative comparison of table 1, where the ROAs, as well as annular dimensions, are compared. In the IMA-AP devices, the ROA is significantly changed with the shortening of the device, while with the IMA-CS there is no substantial change between the three models. Interestingly, the IMA-AP device with 50% had the smallest ROA of all the cases, and further shortening to 70% led to a larger ROA rather than a reduction. Similar to the diastolic configuration of the implantation models, in the systolic configurations the IMA-AP kept the substantial reduction in the AP diameter while this diameter was only slightly changed in the IMA-CS. On the other hand, the IMA-CS is more successful than the IMA-AP in reducing the intercommissural diameter during peak systole, also leading to an annular perimeter reduction from 123.4 to 115 mm, pre- and post-repair in the 22% case, respectively.

**Table 1.** Regurgitant orfice area and anterior–posterior and intercommissural diameters of the mitral valve in peak systolic position.

model	disease	IMA-CS			IMA-AP		
		14%	18%	22%	30%	50%	70%
regurgitant orifice area (mm^2^)	172.8	56.7	55.3	48.2	51.1	27.3	46.1
anterior–posterior diameter (mm)	26.1	25.5	24.7	24.8	20.7	15.9	12.4
intercommissural diameter (mm)	42.9	38.0	37.7	36.8	44.6	46.3	47.9

**Figure 5.**
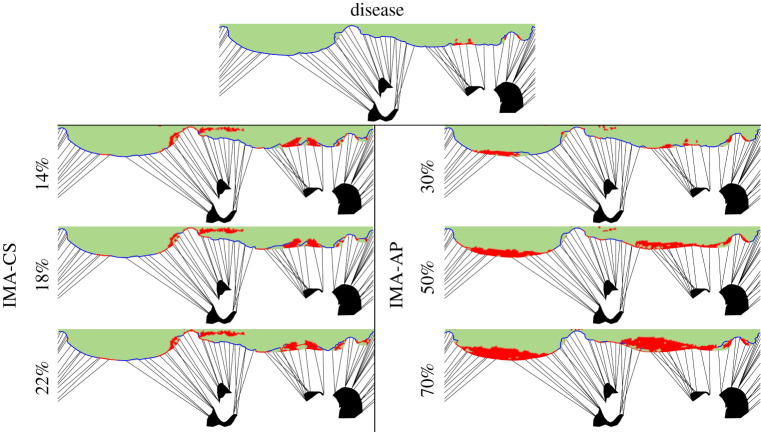
Comparison of spread views of the various cases with marked contact areas (red) and outer contours of the regurgitant orifice areas (blue).

Another way to examine the effectiveness of the treatment is to look at the contact area that represents the coaptation between the leaflets. Figure 5 presents a spread view of the mitral valves in all the cases with red markings for the contact areas. This figure also shows the predicted locations of the leakage, which are marked with blue lines, representing the outer contours of the ROAs shown in figure 4. It can be seen that in the IMA-AP with 50% and 70% shortening models there are much larger contact areas than in the other four repairs. Therefore, it directly indicates a better valve closure because possible leakages can only occur in the gaps between the contact regions. In addition, it can be seen that in the IMA-AP devices the contact region is in the middle of the AP leaflets, while in the IMA-CS there is contact near the commissures and in folds where the posterior leaflet touches itself. These contact regions resemble the contact locations found in the diseased valve, even if they are much larger in the IMA-CS, and they are much less effective at closing the valve. The figure in electronic supplementary material, 3 shows a spread view of the mitral valve in all the cases with contours of the absolute maximum principal strain in peak-systolic position. These results show that the implantation of the devices did not significantly alter the strains in the leaflets. Similarly, the strains in the surrounding tissues that are further away from the implantation site were not significantly affected by the implantations.

### 3.3. Post-implantation function: blood leakage through the valve at peak systole

**Figure 6.**
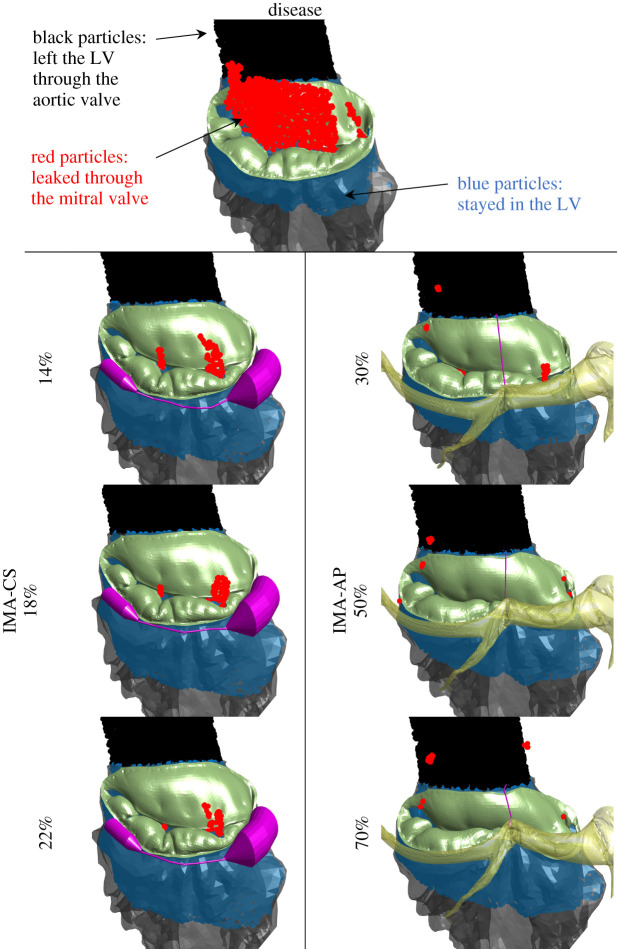
Comparison of the results from the systolic haemodynamic models of the various cases.

In order to obtain a quantitative description of the leakage, the SPH models were used. In figure 6, the colour of the particles represents their locations: red for those that leaked through the mitral valve and reached the LA, black for those that flowed through the aortic valve and reached the aorta, and blue for those that stayed in the LV. Therefore, to quantify the leakage ratio, the number of red particles relative to the total number of red and black particles was calculated. Table 2 compares the calculated leakage ratios from the seven models. All repairs significantly reduced the leakage relative to the disease. The results in table 2 are consistent with those of the ROA that were presented in the previous subsection. In the IMA-AP, the leakage ratio in the 70% model is higher than found in the 50% case, as was also seen in the ROA results. When comparing the two devices, the leakage locations are different. While in the IMA-AP the leakage is close to the commissures, in the IMA-CS the leakage is near the centre of the valve.

**Table 2.** Comparison of the calculated leakage ratio (in percentage) from the various systolic haemodynamic models.

model	disease	IMA-CS			IMA-AP		
		14%	18%	22%	30%	50%	70%
leakage ratio (%)	5.26	0.52	0.41	0.29	0.16	0.08	0.13

## 4. Discussion

In this study, a numerical biomechanics model of a beating heart with FMR was generated and used to examine different generic IMA devices for treatment of the disease. The aim of this study was to compare two types of IMA percutaneous repair techniques, IMA-CS and IMA-AP, by FEA and blood leakage modelling. A total of seven cases, pre-procedural pathology and three different sizes of each device, were examined and compared. To model the IMA treatments, which have not been studied in previous works, the current models were used to evaluate the effect of the device on the systolic function. Since the device is implanted in the relaxed (expanded) heart, the contraction of the heart and its interaction with the devices had to be included to determine the systolic function. This contraction–interaction is another novelty of the current study because this phenomenon could have been ignored in previous models of other devices, such as the MitraClip, where it does not play a major role in the device's function.

From the results of the implantation models, it is clear that although both types of IMA devices are considered ‘indirect’, the IMA-AP device has a much more direct effect on the mitral annulus than the IMA-CS. While it might not be a direct reduction of the annular perimeter or the intercommissural diameter, it has an almost direct effect on the AP diameter. This effect can be demonstrated by the fact that almost all the reduction in the device's length is translated into a shortening of the annular diameter. For example, in the case of IMA-AP 70%, shortening of 22.7 mm led to a reduction of 20.1 mm in the AP diameter. On the other hand, in the IMA-CS device, which is located further away from the mitral annulus, the reduction of the device length has a truly indirect effect on the annulus. For example, a reduction of 6.7 mm in the length of the bridge of the IMA-CS 22% case led to a reduction of only 1.8 mm in the annular intercommissural diameter. Still, even this small change proved to have a significant effect on the regurgitation, as was found from the following simulations of the heart contraction and the systolic blood flow.

From the results of the IMA-AP, both the ROA and the leakage ratio that was calculated with the SPH models, small leakages were formed in the commissure region. Counterintuitively, both the leakage ratio and the ROA increase when the AP diameter is further shortened from the 50% to the 70% case. The 70% case led to a better closure near the device, in the middle of the leaflets, but it also opened the valve on the sides of the leaflets. Perhaps IMA-AP shortening in two or more locations, like suggested in the TASRA device [43], can help prevent this phenomenon and form a more homogeneous closure across the valve. It is also interesting that the location of the leakage in the IMA-AP seems to conform with the location of the leakage shown in the studies of the MitraClip [32,36], demonstrating that it is a result of central grasping. Moreover, like in the IMA-AP device, in the IMA-CS the contact was also near the anchors of the device, shifting the leakage to the middle in this case. Therefore, while the location of the leakage was not considered as a success criterion in the current study, it demonstrated how the selection of device type can control it.

The models produced additional interesting results, such as the mechanical response of the devices. For example, in the three models of the IMA-CS devices, the alternating strain amplitudes in the cyclically deforming bridges were less than 0.125%. While the modelled devices are merely generic, these amplitudes are still markedly lower than the endurance limit of NiTi, which was previously suggested to be 0.4% [44]. Although these results indicate that the IMA-CS devices are expected to successfully pass 4×10^8^ cycles [44], it should be emphasized that our current calculations were done for a generic device and were not meant to be used for fatigue estimation.

While in this study, we were able to model the function of two IMA devices that have never been modelled before, there are some limitations to the simulations. First, in the IMA-CS device, the implantation was modelled inside the CS vein, but in the beating heart model the anchors of the device were directly ‘tied’ to the LV and LA for simplification. This simplification is reasonable in the current models with generic devices because we only tried to capture the effect of the annular reshaping on the valve function and because the CS vein itself is connected to the LV and LA. In future models, we plan to introduce a device design that better resembles the commercial IMA-CS, which will be modelled inside the CS vein throughout the cycle. It will also enable us to account for additional phenomena like fatigue estimation, as explained above, and possible circumflex artery compression. In the IMA-AP models, the suture was connected directly to the CS and LA for simplification. In future studies, we plan to model the implantation of the device in a more realistic way by considering the shape of the two anchors that are connected by the suture. Another limitation is related to the SPH model, where the geometry was based on the peak systolic position rather than modelling fluid-structure interaction. This simplification was done for a more direct comparison with the geometric ROA calculation, and it will be modelled with such a coupling in future models that will focus on the haemodynamic itself. In this study, we focused on the most important choice the interventional cardiologists need to make during IMA procedure, the distance between the anchors if both anchors are implanted in their recommended locations. Still, any variation in the implantation location might have a significant impact on the procedural results, and this impact can be evaluated in future studies. Finally, although this is not a patient-specific model, we compared several cases and generic devices and presented the results in a comparative manner only. For this reason, we searched for trends that should be relevant for any device that is based on the two IMA techniques, instead of searching for specific repair recommendations. Future studies can expand these attempts to various patient-specific pathological conditions to give a more precise procedural recommendation.

In summary, we introduced numerical biomechanics models of implantations of two IMA devices and their effect on MR in the LHHM. The aim was to examine the reduction in leakage and the improvement in valve coaptation after those repairs. The implantation of the IMA-AP devices led to better coaptation relative to the IMA-CS devices. In the IMA-AP devices, the optimal ROA and leakage ratio were found in the medium shortening, probably because the shortest device enlarged the openings in the commissures' region. On the other hand, in the IMA-CS device, there is a direct correlation between the length of the device and the reduction in the leakage. These findings can help in bringing IMA-AP to market, expanding the use of IMA devices in general, and optimizing future designs of such devices.

## Data accessibility

Our dataset is available from the Dryad Digital Repository: https://doi.org/10.5061/dryad.bzkh1899d [45].
